# Intraoperative radiotherapy (IORT) is an option for patients with localized breast recurrences after previous external-beam radiotherapy

**DOI:** 10.1186/1471-2407-7-178

**Published:** 2007-09-14

**Authors:** Uta Kraus-Tiefenbacher, Lelia Bauer, Antonella Scheda, Carola Schoeber, Joerg Schaefer, Volker Steil, Frederik Wenz

**Affiliations:** 1 Department of Radiation Oncology, Mannheim Medical Center, University of Heidelberg, Theodor-Kutzer Ufer 1-3, D-68167 Mannheim, Germany; 2 Department of Gynecology and Obstetrics, Mannheim Medical Center, University of Heidelberg, Theodor-Kutzer Ufer 1-3, D-68167 Mannheim, Germany; 3 Department of Clinical Radiology, Mannheim Medical Center, University of Heidelberg, Theodor-Kutzer Ufer 1-3, D-68167 Mannheim, Germany

## Abstract

**Background:**

For patients suffering of recurrent breast cancer within the irradiated breast, generally mastectomy is recommended. The normal tissue tolerance does not permit a second full-dose course of radiotherapy to the entire breast after a second breast-conserving surgery (BCS). A novel option is to treat these patients with partial breast irradiation (PBI). This approach is based on the hypothesis that re-irradiation of a limited volume will be effective and result in an acceptable frequency of side effects. The following report presents a single center experience with intraoperative radiotherapy (IORT) during excision of recurrent breast cancer in the previously irradiated breast.

**Methods:**

Between 4/02 and 11/06, 15 patients were treated for in-breast recurrences at a median of 10 years (3–25) after previous EBRT (10 recurrences in the initial tumor bed, 3 elsewhere in-breast failures, 2 invasive recurrences after previous DCIS). Additional 2 patients were selected for IORT with new primary breast cancer after previous partial breast EBRT for treatment of Hodgkin's disease. IORT with a single dose of 14.7 – 20 Gy 50 kV X-rays at the applicator surface was delivered with the Intrabeam™-device (Carl Zeiss, Oberkochen, Germany).

**Results:**

After a median follow-up of 26 months (1–60), no local recurrence occurred. 14 out of 17 patients are alive and free of disease progression. Two patients are alive with distant metastases. One patient died 26 months after BCS/IORT due to pulmonary metastases diagnosed 19 months after BCS/IORT. Acute toxicity after IORT was mild with no Grade 3/4 toxicities and cosmetic outcome showed excellent/good/fair results in 7/7/3 cases.

**Conclusion:**

IORT for recurrent breast cancer using low energy X-rays is a valuable option for patients with recurrent breast cancer after previous radiotherapy.

## Background

External-beam radiotherapy (EBRT) of the breast after breast conserving surgery (BCS) reduces the local breast tumour recurrence rate from 25 – 30% to less than 10% at 10 years [1-6]. However, it is still a problem to find the optimal therapy modality for the remaining 10% of breast cancer patients presenting with a tumour recurrence years after BCS and EBRT. The normal tissue tolerance does not allow, even after years, a second full-dose course of radiotherapy to the entire breast after a second BCS. Especially for patients with small, localized recurrences, in whom a local excision would technically be possible, mastectomy is generally preferred over BCS for fear of worse outcome due to omission of radiotherapy. This is particularly unsatisfying, because recurrent breast tumours, with increasing advances in diagnostic modalities and regular follow-up visits, are often diagnosed at a very small tumour size. Furthermore, the most common and survival-limiting problem for these patients is usually not the local situation within the breast, but the increased risk of developing distant metastases [7]. Finally, more than 90% of all IBTRs occur in the vicinity of the index tumour [8,9].

A novel option is to treat these patients after re-resection of the recurrent tumor with partial breast irradiation (PBI). This approach is based on the hypothesis that re-irradiation to a limited volume will be effective and result in an acceptable frequency of side effects. Intraoperative radiotherapy is one option to deliver high doses to a restricted area at risk i.e. the adjacent tissue to the tumour cavity after tumour resection. IORT can be delivered with dedicated linear accelerators in the operation room or novel mobile devices using electrons or low-energy x-rays [e.g. [10,11]].

The following report presents a single center experience with intraoperative radiotherapy (IORT) to a partial breast volume during excision of recurrent breast cancer in a previously irradiated breast. The technical and radiobiological aspects of this treatment method using low-energy soft X-rays were described in detail previously [12-15]. KV X-rays are amongst others characterized by a sharp dose fall-off to the tissue depth. If a dose of 20 Gy is prescribed to the applicator's surface for example, the dose in 1.0 cm is, depending on the applicator size, only 5 Gy, but due to their different radiobiologic behaviour, a comparison between the dose delivered by photons/electrons or soft X-rays is not approvable.

## Methods

Included in this analysis were all 17 patients with breast cancer in a previously irradiated breast who presented consecutively in the interdisciplinary tumourboard at the Mannheim Medical Center/University of Heidelberg between 4/2002 and 11/2006. The medical ethics committee II (University of Heidelberg) stated that ethics approval is not needed for this retrospective analysis.

Median age was 65.8 years (48.3–86.9). All patients had previous EBRT to the breast, 13 due to primary breast cancer, 2 due to DCIS and 2 due to EBRT of the mediastinal and axillary lymph nodes because of Hodgkin's disease.

**Figure 1:**
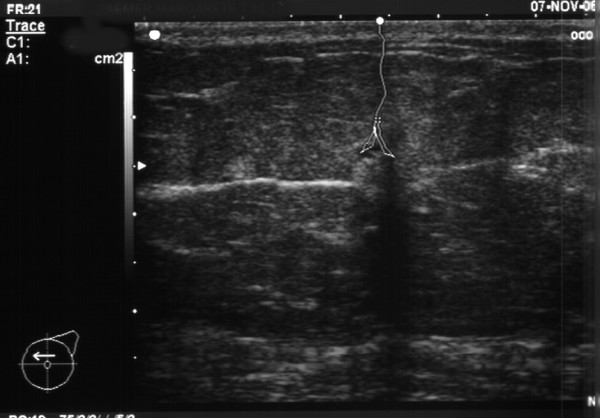
Breast-ultrasound for routine follow-up of a patient (no.17, 16 years after primary breast cancer) showed a small hypoechogeneity within the previous index quadrant.

**Figure 2:**
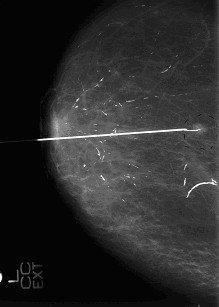
Craniocaudal mammography of the same patient with a small dense structure suspicious for recurrent breast cancer. This non-palpable lesion was marked by a wire before surgery.

**Figure 3:**
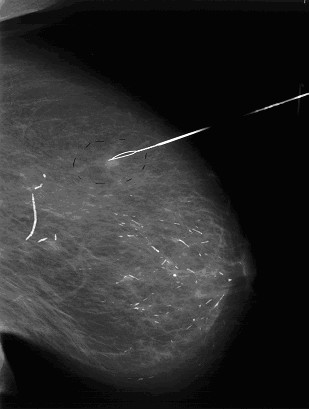
Oblique mammography of the same patient.

For previous treatment and exact total delivered dose, see patient characteristics in Table 1. 10/17 patients had the same histology and tumour localization (same quadrant) as their primary breast cancer (true in-breast tumour recurrences, IBTR, for a representative patient example see figures 1, 2, 3), 3/17 presented with recurrences with a different histology, but the same localization and were categorized as secondary breast cancers. 2/17 had previous EBRT due to DCIS and developed invasive recurrences during further follow-up. Finally 2/17 patients developed invasive breast cancer after treatment for Hodgkin's disease years before. Median duration between EBRT for primary and IORT for recurrent disease was 10 years (3–25) and median recurrent tumour size was 11 mm (1–35 mm). At time of IORT, the exact status of the margins was not known, but specimen-x-rays were done in any case and could show the complete resected tumours. Definitive pathology results showed afterwards a median margin size of 3 mm with a range between 1 and 10 mm. IORT was delivered with the Intrabeam™-device (Carl Zeiss, Oberkochen, Germany). This system includes several spherical applicators ranging from 1.5 to 5.0 cm in diameter, resulting in an isotropic dose-distribution of 50 kV X-rays with a very sharp dose-fall off. This device has been used in our institution since 2/2002 in more than 220 breast cancer cases. The median applicator size used for these 17 patients was 4.0 cm (2.5–5.0), median dose at the applicator surface was 20.0 Gy (14.7–20.0) and median treament time was 26.8 minutes (18.6–35.9). In addition, 3/17 patients were treated by chemotherapy and 14/17 by hormonal therapy. Exact type of systemic therapies is shown in Table 2. All patients underwent a prospective, predefined follow-up including clinical examination and breast-ultrasound at 6 monthly and mammographies at yearly intervals. Toxicities were documented using the CTC/EORTC- and the LENT SOMA-score. Cosmesis was evaluated with a 1–4 score as previously published [15].

**Table 1:** Patient characteristics part I

Pat.No.	Age *	Type of recurrence**	Years after primary disease	Histology primary disease	Histology recurrent disease	Local therapy of primary disease
1	53.9	IBTR	3	lob-inv	lob-inv	BCS + 56 Gy
2	67.2	IBTR	3	duct-inv	duct-inv	BCS + 56 Gy
3	52.1	SBC.	4	duct-inv	aden-cyst	BCS + 62 Gy
4	65.8	SBC	9	lob-inv	duct-inv	BCS + 56 Gy
5	80.7	IBTR	11	lob-inv	lob-inv	BCS + 56 Gy
6	70.6	IBTR	4	duct-inv	duct-inv	BCS + 49 Gy
7	74.1	IBTR	9	lob-inv	lob-inv	BCS + 56 Gy
8	53.9	SBC	13	lob-inv	duct-inv	BCS + 56 Gy
9	48.3	IBTR	14	duct-inv	duct-inv	BCS + 50 Gy
10	75.8	IBTR	17	duct-inv	duct-inv	BCS + 56 Gy
11	78.0	IBIR	10	DCIS	duct-inv	BCS + 56 Gy
12	60.8	IBTR	14	muc-inv	muc-inv	BCS + 56 Gy
13	58.0	IBTR	7	duct-inv	duct-inv	BCS + 56 Gy
14	58.5	IR	10	DCIS	duct-inv	BCS + 56 Gy
15	86.9	IBTR	16	duct-inv	duct-inv	BCS + 56 Gy
16	56,8	BC after Hodgkin	5	nodular	duct-inv	Med 30 Gy
17	66.2	BC after Hodgkin	25	nodular	duct-lob-inv	Med 40 Gy

* Age at time of IBTR, ** Different types of recurrences:

IBTR = In-breast tumour recurrences (which have the same histology and localization)

SBC = Secondary breast cancer (which show a different histology)

IR = Invasive recurrences (after previous DCIS)

BC after Hodgkin = invasive breast cancer after Hodgkin's disease.

Histologies: Duct-inv = ductal-invasive, lob-inv = lobular-invasive, DCIS = Ductal carcinoma in situ, muc-inv = mucinous-invasive, nodular = nodular Hodgkin's disease, duct-lob-inv = mixed histology, partly ductal-invasive, partly lobular-invasive.

Local therapy of primary disease: BCS = breast conserving surgery

Med 30/40 Gy = the two patients with Hodgkin's disease were treated by involved field radiotherapy to the mediastinal lymph nodes with 30/40 Gy respectively.

**Table 2:** Patient characteristics part II:

Pat.No.	Systemic therapy of primary disease	Systemic therapy of secondary disease	Recurrent tumour size (mm)	Applicator size IORT (cm)	Dose IORT (Gy)	Treatment time IORT (Min)
1	GnRH	Tamoxifen	12	3.5	20,0	18,60
2	6 × CMF	AI	35	5.0	14,7	35,90
3	None	GnRH	1	3.5	20,0	18,60
4	Tamoxifen	AI	16	4.5	14,7	28,70
5	Tamoxifen	AI	16	4.5	14,7	28,70
6	Tamoxifen	AI	12	4.0	20,0	26,80
7	None	AI	8	3.0	20,0	24,98
8	None	Tamoxifen	7	4.0	20,0	26,80
9	6× CMF + Tam	AI	15	4.0	20,0	26,80
10	3× CMF + Tam	AI	18	4.0	20,0	26,80
11	None	Tam	10	4.0	20,0	28,26
12	None	AI	9	3.5	20,0	19,9
13	None	none	11	5.0	20,0	49,5
14	None	none	11	3.5	20,0	19,9
15	4× EC + Tam	AI + GnRH	11	3.0	20,0	26,4
16	4× ABVD	leftTamoxifen	11	2.5	20,0	19,2
17	none	4 × FEC	11	5.0	20	49.50

GnRH = Gonadotropin Releasing-Hormone Analoga, Tam = Tamoxifen, CMF = Cyclophosphamide (600 mg/m^2^)/Methotrexate (40 mg/m^2^)/5-Fluorouracil (600 mg/m^2^)

EC = Epirubicin (90 mg/m^2^)/Cyclophosphamide (600 mg/m^2^), AI = Aromatase inhibitors

ABVD = Adriamycin, Bleomycin, Vinblastin, DTIC

## Results

After a median follow-up of 26 months (1–60), 16 out of 17 patients are still alive. One patient (no.3) with secondary breast cancer with an uncommon adenoid-cystic breast tumour (primary breast cancer 4 years before has been ductal-invasive) died 26 months after BCS/IORT due to pulmonary metastases that were diagnosed 19 months after treatment for recurrence. Two other patients had distant metastases (retroorbital and bone) before and after treatment of their primary breast cancer. All localizations were treated by palliative EBRT and systemic treatment with bisphosphonates. Both patients are still alive in good overall condition.

Acute toxicity after IORT was mild with no Grade 3/4 toxicities and there was no delay in wound healing nor any wound infection. 6/17 patients (35.3%) showed a limited induration of the tumourbed. In 3/17 (17.6%) cases the induration of the area treated with IORT was judged as moderate to severe.

The cosmetic outcome after a median follow-up of 26 months was satisfactory with excellent/good/fair results in 7/7/3 cases. All 3 patients with only fair cosmetic results had already small breast volumes after previous BCS and EBRT and developed a moderate/severe induration of the remaining breast tissue after second intervention, which resulted in a suboptimal cosmetic outcome.

## Discussion

Mastectomy is the current standard of care for IBTR of breast carcinoma, but there are some reasonable arguments for a second breast-conserving approach:

1. The risk of local recurrence is not eliminated by using mastectomy. Approximately 2–32% of patients treated with mastectomy for recurrent breast cancer develop a chest wall recurrence [17].

2. In the NSABP-B06 protocol, IBTR was an independent predictor of distant disease with an adjusted relative risk of 3.41 [7]. A previously published analysis comparing the data of 2669 patients from 5 NSABP node positive protocols (B15, 16, 18, 22 and 25) confirmed these results and found that 5 years after IBTR, only 51.4% of the patients were free of distant disease [18]. The time prior to IBTR has been proposed to be an additional prognostic factor for survival. If IBTR occurred within 5 years of initial therapy, the 5-year overall-survival rate and the 5-year distant-recurrence-free survival rate was 65% and 61% respectively, compared to patients with IBTR 5 or more years after initial diagnosis showing an overall-survival rate of 81% and a 5-year distant-recurrence-free survival rate of 80% [19]. Consequently, the spread of distant tumour cells seems to be the survival-limiting problem of patients with IBTR, not the risk of re-recurrences within the breast. Recurrences elsewhere in the ipsilateral breast or second primaries may have a completely different behaviour, because theoretically these tumours represent de novo disease rather than persistent, radioresistant, drug-insensitive disease [18].

There are a few reports about acceptable long-term control rates after BCS with or without additional radiotherapy:

52/118 patients with IBTR after BCS and EBRT for stage I or II breast cancer were selected for salvage treatment with wide excision, with or without axillary dissection, instead of mastectomy [20]. With a median follow-up of 6 years, the actuarial cancer-specific survival after treatment of recurrence was 79% at 5 years and 64% at 10 years. The local control rate in the treated breast was 79% at 5 years. Of 12 patients in whom second local or regional recurrences developed, ten could be treated by further surgery. The authors concluded that wide excision represents an adequate alternative to mastectomy in the salvage treatment of isolated breast recurrences at least for selected patients (mobile tumours, 2 cm or smaller in diameter, no signs of rapid growth) [20]. The same investigator group reported the feasibility of conservative salvage surgery in a clinicopathologic study 3 years later. After wide excision for 50 selected patients with IBTR after standard BCS, 16 (32%) second local failures occurred after a median follow-up of 51 months (5-year local control 62%). A Cox multivariate analysis of 18 parameters indicated that only disease-free interval and resection margins significantly influenced local control. 5-year local control was 92% for recurrences occurring after 5 years vs. 49% for shorter intervals, and 73% for negative vs. 36% for positive or indeterminate margins. Local control appeared to be independent of morphologic features, initial tumour stage, patient age, recurrent tumour size and location. Median survival after second local failure was 33 months. Particularly for late recurrences, wide excision with negative margins seems to be a satisfactory alternative to mastectomy [21]. Another report on 16 patients with IBTR after BCS and EBRT (median interval between first BCS and IBTR 31 months) exists, who refused mastectomy and were treated with repeat lumpectomy and radiotherapy. 15 patients were treated with a total dose of 50 Gy and a fractional dose of 2 Gy and 1 patient with a total dose of 32 Gy and a fractional dose of 2 Gy to the tumourbed. After a follow-up between 42 and 119 months, 4 patients had further local failure (2 of them additionally distant metastases), 10 patients are alive and free of disease and 2 patients developed distant metastases. The authors concluded that a repeat course of radiotherapy for an IBTR for selected patients is well tolerated without any severe late sequelae and may provide long-term local control [22]. The same group again evaluated the outcome of another 23 patients with an IBTR (median interval between first BCS and IBTR 63 months) after lumpectomy and breast irradiation for invasive carcinoma (n = 31) or ductal carcinoma in situ (n = 8), who were treated with excision of the IBTR and EBRT of the tumour bed with 50 Gy in 25 fractions using electrons of appropriate energy. Again, the second course of EBRT was well tolerated in all patients with no other late sequelae than skin pigmentation changes. 30 patients (76.9%) had an intact breast free of tumor at death or at last follow-up 1–180 months (median 51.5) after reirradiation and 8 patients developed distant metastases. The estimated overall and disease-free 5-year survival rate for the 39 patients was 77.9% and 68.5%, respectively. The conclusion was drawn that the excision of the IBTR followed by repeat external beam RT to the operative area may be an acceptable alternative to mastectomy for selected patients [23]. Early follow-up studies of breast reirradiation suggest also that beside standard external beam radiation therapy, catheter-based interstitial brachytherapy can be delivered to the breast more than once without inacceptable side effects in most patients. with acceptable cosmesis in some patients. Mastectomy may not be necessary in all patients with an in-breast local recurrence of breast carcinoma [17]. 69 patients with IBTR after previous lumpectomy and radiotherapy were treated in France by second breast conserving surgery and interstitial brachytherapy with a total dose of either 30 (n = 24) or 45–50 Gy (n = 45). Catheters were implanted in all cases immediately after tumour resection and radiotherapy started 5 days after surgery. Five-year disease free survival after first local recurrence was 77%. After a median follow-up of 50 months, up to 22% grade 2 and 3 long term side-effects like fibrosis, breast retraction or teleangiectasia occurred [24].

Comparing the results of the above mentioned studies to our data, regarding toxicity of the reirradiated patients, several parallels could be identified, particularly with the latter brachytherapy study. One advantage of the reirradiation with IORT compared to interstitial brachytherapy, where patients have to be irradiated over several days, is certainly the time-saving single day treatment.

The role of additional systemic therapy in the management of IBTR remains uncertain. Although the use of hormonal therapy has been shown to be effective [25,26], the benefit of chemotherapy has not been adequately evaluated in prospective randomized trials [18].

A limited number of reports about the use of radiotherapy for breast cancer after irradiation of Hodgkin's disease has been published. The group from Milan [27,28] reported a lack of acute complications and good cosmetic results in 6 patients undergoing IORT during BCS years after Mantle field irradiation. Deutsch et al [29] described 12 patients treated with full-dose EBRT after BCS for breast cancer in previously irradiated breasts following radiotherapy for Hodgkin's disease.

## Conclusion

This is to our knowledge the first report about IORT using 50 kV X-rays for recurrent breast cancer in a previously irradiated breast tissue. No unexpected toxicities were observed during a median follow-up of more than 2 years. IORT of the tumourbed may become an alternative to mastectomy for selected patients with an IBTR after previous breast irradiation.

## Competing interests

The author(s) declare that they have no competing interests.

## Authors' contributions

UKT was involved in patient selection and follow-up, coordination of the procedures, initial drafting and writing of the manuscript. LB was involved in patient selection and follow-up, performed all surgeries, received informed consent for surgery. AS was involved in patient follow-up, clinical assessment and data analysis, received informed consent for radiotherapy. CS was responsible for detection and diagnostic work-up before and after surgery, participated in data analysis. JS was involved in patient follow-up, clinical assessment and data analysis, editing of the manuscript. VS was responsible for quality assurance, all medical physics aspects, and dosimetry, proof-reading of manuscript. FW was responsible for design and conception, involved in patient selection, responsible for final data analysis, responsible for writing and finalizing of the manuscript. All authors read and approved the final manuscript.

## Pre-publication history

The pre-publication history for this paper can be accessed here:


